# Why do we use transpulmonary thermodilution and pulmonary artery catheter in severe shock patients?

**DOI:** 10.1186/s13613-024-01400-4

**Published:** 2025-01-14

**Authors:** Xavier Monnet, Christopher Lai, Daniel De Backer

**Affiliations:** 1 AP-HP, Service de Médecine Intensive-Réanimation, Hôpital de Bicêtre, DMU 4 CORREVE, Inserm UMR S_999, FHU SEPSIS, CARMAS, Université Paris-Saclay, 78 Rue du Général Leclerc, 94270 Le Kremlin-Bicêtre, France; 2 Department of Intensive Care, CHIREC Hospitals, Université Libre de Bruxelles, Brussels, Belgium

## Introduction

Although cardiac output (CO) is a main actor in acute circulatory failure and its monitoring is recommended by international consensus in severe patients with shock who resist to initial treatment [1], not all intensivists do so. Why is it useful to measure CO in such patients? Why should one use devices that measure CO continuously, like transpulmonary thermodilution (TPTD) and the pulmonary artery catheter (PAC)? Although less used than before, PAC keeps an added value in selected indications [2], but what are these indications? In this position article, we provide some answers to these questions.

## Knowing cardiac output is helpful when managing shock

CO is crucial for delivering oxygen to tissues, making it a key variable to monitor during shock. Measuring CO helps identify the type of shock. In hypovolemic and cardiogenic shock, CO is typically low, while CO tends to be elevated in septic shock, especially after initial fluid resuscitation [3]. The information beyond CO provided by PAC and TPTD, especially regarding cardiac preload or contractility, also helps characterizing the haemodynamic failure.

Measuring CO is essential for assessing the effectiveness of treatments like fluids and inotropes. Their therapeutic margin is low. In such a life-threatening condition, a rough estimate of their effectiveness is unreasonable. For instance, monitoring CO allows evaluation of the response to a fluid bolus and, in the case of unresponsiveness, discontinuation of fluid resuscitation. Similarly, clinicians cannot accurately assess inotropes efficacy without knowing their effect on CO.

## Cardiac output should be directly measured

At the bedside, there is no way to perfectly measure CO. However, the worst estimate comes from surrogates, which seek to estimate CO and its changes by observing peripheral haemodynamic variables. For example, diuresis poorly correlates with changes in CO following volume expansion [4]. Changes in heart rate also unreliably assess fluid responsiveness, even for extreme changes [5].

More commonly, arterial pressure is used to judge the effects of volume expansion or inotropes on CO. However, it also depends on arterial resistance and compliance. Pulse pressure changes are poorly [5, 6] or not at all [7] correlated with fluid-induced changes in CO, even less when norepinephrine doses are modified [6]. Arterial pressure only is not enough for monitoring severe patients with circulatory failure.

## Only dedicated devices allow real cardiac output monitoring

**Fig. 1:**
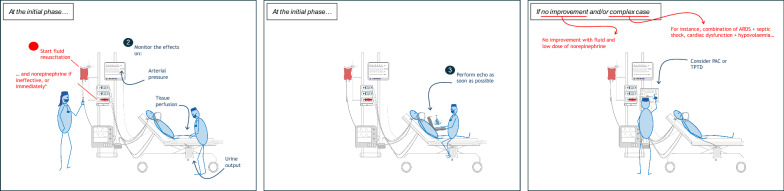
Sequential haemodynamic assessment in patients with shock

Echocardiography is essential for assessing cardiac function and structure and must be performed initially in shock patients (Fig. 1). However, it is usually performed only once or twice on the first day of treatment. Repeating it more often, i.e., at each haemodynamic assessment, for each preload responsiveness test, before and after each volume expansion or change in inotrope dose is time-consuming and hardly feasible.

## Advanced techniques provide global haemodynamic evaluation

TPTD and PAC reliably estimate CO in most conditions, although they are not perfect. Less invasive devices are likely less reliable. For instance, uncalibrated pulse contour analysis fails to estimate CO when arterial resistance changes [8], especially under norepinephrine [9], though it may track CO changes during fluid infusion or tests of preload responsiveness.

The advantage of TPTD and PAC is that they offer more than just CO measurements. TPTD measures extravascular lung water and pulmonary vascular permeability, which guide fluid therapy, especially in acute respiratory distress syndrome (ARDS). They predict outcome [10] and lung water reflects diffuse alveolar damage [11]. TPTD estimates cardiac contractility [12] and measures CO continuously by calibrated pulse wave analysis, allowing an easy assessment of preload responsiveness [13, 14].

PAC remains a reliable tool that provides comprehensive haemodynamic information [15]. It provides the only reliable estimate of left atrial pressure [16]. It measures pulmonary vascular resistance and so is valuable in right heart failure and ARDS, for setting positive end-expiratory pressure, for instance [17]. PAC also assesses the true values of mixed venous oxygen saturation and carbon dioxide partial pressure [15], which are crucial for evaluating tissue wellness.

## For a reasoned use of advanced haemodynamic monitoring tools

Haemodynamic monitoring cannot be based only on CO measurements and knowing CO in isolation is not enough for taking decisions. For instance, indices of fluid tolerance also influence the decision to give fluid. TPTD and PAC interestingly provide such variables in addition to CO. Also, the goal is to improve tissue perfusion and oxygenation, not CO. However, without knowing CO, indices of tissue perfusion and cell metabolism cannot be interpreted. For instance, if capillary refill time does not improve after volume expansion, it could mean that CO has not changed due to preload unresponsiveness, or that CO has increased but microcirculation remains impaired [18].

Due to their cost and invasiveness, TPTD and PAC should be reserved for the most severe cases of shock, particularly in patients with complex conditions like sepsis, cardiac dysfunction, ARDS, or intra-abdominal hypertension, when shock resists initial treatment with fluid and low-dose vasopressors (Fig. 1). In these patients, additional haemodynamic information provided by advanced monitoring are particularly helpful. Although many devices estimate and continuously measure CO, PAC and TPTD devices measure additional important haemodynamic variables often not available otherwise, which make these attractive if used in a logical and effective manner. Of course, the choice of which device to use depends on the clinical situation and the intensivist’s experience with each technique (Fig. 1).

There is an ongoing debate about whether using advanced monitoring techniques reduces mortality. While early studies failed to show a clear survival benefit, more recent research suggests that PAC may improve outcomes in patients with cardiogenic shock [19]. In septic shock, demonstrating a survival benefit is more challenging due to the complexity of constructing a treatment algorithm that works for all patients (20). However, future studies may show that strategies targeting specific haemodynamic variables—provided by advanced monitoring systems—can improve patient outcomes, especially if they use relevant endpoints other than mortality.

## Conclusion

In the most severe and complex shock patients, efficient management requires a complete view of haemodynamic status, including CO. In our opinion, only TPTD and PAC reliably measure CO in such patients and provide sufficient haemodynamic information to guide their treatment.

## Data Availability

Not applicable.
